# Tracking of Ball and Players in Beach Volleyball Videos

**DOI:** 10.1371/journal.pone.0111730

**Published:** 2014-11-26

**Authors:** Gabriel Gomez, Patricia Herrera López, Daniel Link, Bjoern Eskofier

**Affiliations:** 1 Digital Sports Group, Pattern Recognition Lab, Friedrich-Alexander Universität Erlangen-Nürnberg, Erlangen, Germany; 2 Departamento de Ingeniería de Telecomunicación, Universidad Politécnica de Madrid, Madrid, Spain; 3 Department of Training Science and Sport Informatics, Technische Universität München, Munich, Germany; University of Chinese Academy of Sciences, China

## Abstract

This paper presents methods for the determination of players' positions and contact time points by tracking the players and the ball in beach volleyball videos. Two player tracking methods are compared, a classical particle filter and a rigid grid integral histogram tracker. Due to mutual occlusion of the players and the camera perspective, results are best for the front players, with 74,6% and 82,6% of correctly tracked frames for the particle method and the integral histogram method, respectively. Results suggest an improved robustness against player confusion between different particle sets when tracking with a rigid grid approach. Faster processing and less player confusions make this method superior to the classical particle filter. Two different ball tracking methods are used that detect ball candidates from movement difference images using a background subtraction algorithm. Ball trajectories are estimated and interpolated from parabolic flight equations. The tracking accuracy of the ball is 54,2% for the trajectory growth method and 42,1% for the Hough line detection method. Tracking results of over 90% from the literature could not be confirmed. Ball contact frames were estimated from parabolic trajectory intersection, resulting in 48,9% of correctly estimated ball contact points.

## Introduction

With increasing computational power of modern computers, complex tracking algorithms are becoming more feasible even for real time applications. Even in offline applications, time efficiency can be an important issue. In the case of professional sports, when doing e.g. video analysis of matches during a tournament, it is important that the material needed by the trainers be available as fast as possible after a match, be it the own team or an opponent being analyzed. While there are several costly and complex methods on the market for video analysis using multiple cameras in popular sports like soccer or basketball, some sports still struggle with inefficient information gathering such as by manual annotation. The methods presented in this paper were implemented to make video analysis more efficient for beach volleyball, allowing more videos to be analyzed in less time and thus giving more accurate statistics for an optimal technical or tactical preparation by the trainer staff. The methods were implemented in a software called BeachTracker, which is one of three custom made tools used for the German Volleyball Federation in order to support the German National Teams. Although developed for the challenging situation that beach volleyball sets to tracking, such as mutual occlusion of the players, net occlusion, sunny and shadowy regions, sand structure and color similar to human skin, the presented methods can be applied to other sports or surveillance applications as well with little modifications.

## Literature Overview

Object tracking has become one of the most important fields in computer vision, going hand in hand with automation processes in industry (e.g. automatically finding flaws in production parts), surveillance, traffic monitoring, forgery detection, robotics, medical procedures, sport analysis, etc. Depending on the object to be tracked and on the scene conditions, different tracking methods can be applied to optimize the results, such as blob tracking, kernel based tracking, contour tracking and the Bayesian approach, used in this work. In blob detection, regions in an image are sought that differ from the rest. This can be done with template matching [1], automatic scale detection [2] or optical flow [3], to name a few. In kernel based tracking, the kernel, a rectangular or elliptical region in an image, is defined and its motion calculated. Translation, rotation or an affine transformation of that kernel between frames is taken into account [4]. In contour tracking, the boundaries of an object are detected. Two prominent methods are the active contour method defined in [5] and the fast radial symmetry transform [6]. In Bayesian estimation, the state of a system and its measurement are defined. Throughout consecutive frames, the state of the system (e.g. the position of the tracked object) is predicted using previous state information and new measurements. A good introduction to a prominent method, the Kalman Filter, is presented in [7]. The particle filter [8] and the Markov Chain Monte Carlo [9] are two other widely used Bayesian methods. In recent years, there has also been the approach to use multiple tracker methods that work separately and combine the outputs to a final more robust tracking result [10].

In professional sports, computer vision has become an important tool for game analysis. The main goal here is to extract useful information such as statistics on player interactions, efficiency of the players, direction of a ball hit or other information related to the specific sports type. Due to its simple yet powerful algorithm, particle filtering is one of the mostly used approaches in object tracking in sports. Tracking of the players with the use of particle filters has been proposed in [11] for soccer. Players in basketball and squash [12] were also tracked with particle filters in combination with rules and assumptions related to the position of the camera and the players. In beach volleyball, the works of [13], [14] respectively use particle filters combined with blob detection or segmentation algorithms. In the case of beach volleyball, in both works the background model was created in the first step. In [13], color information was then used in an integral histogram approach that also allows rotational movement tracking of the front players. Here, only the torsos and upper legs were used for the color based tracking, while a segmentation step later searched for the feet of the players using a trained skin model. The predicted state of the particle set was the position on the field, velocity and rotation angle of the torsos. In [14] on the other hand, color histograms and motion information of the players from a background subtraction algorithm were used to track the players both in the front and back of the field. The particle state being the position of the players on the field in world coordinates was estimated directly using the whole body of the players rather than segmenting the feet in a second processing step. As it is very difficult to define the position of the players on the field while they are jumping (wrong homographic transformation estimates) or with their torsos rotated (the position on the field could be defined as either between the feet or below the center of gravity, for example), rotation was not taken into account. Also, due to the nature of the game, the players and their limbs experience very fast movements often changing their direction abruptly, making a velocity dependent resampling often disadvantageous. Therefore, the position of the particles as a state (with an optimized random noising in the resampling process) was found sufficient for the task.

## Methods

The work presented in this paper is an extension of the works by [13] and [14]. It goes further by first using a novel foreground extraction algorithm combined with a background subtraction algorithm that updates itself to compensate for scene illumination changes. Second, it uses several different cues that combined give a total weight to the tracker's particles. Third, a rigid grid approach is introduced with particles positioned at static distances inside the grid. Last of all, an existing and a new algorithm for tracking the ball in beach volleyball videos are explained and compared to each other. All algorithms are explained in detail in the next sections. We start by explaining the methods used for the tracking of the players in section 3.1. The state probability of the players (the estimated position in world coordinates) is calculated based on three different cues extracted from each frame. The cues are the movement of the players, their individual color information and the foreground probability. Then we describe the tracking of the ball in section 3.2, where some of the methods from section 3.1 are used as well.

### 1. Tracking of the players

#### a) Homographic transformation

**Figure 1:**
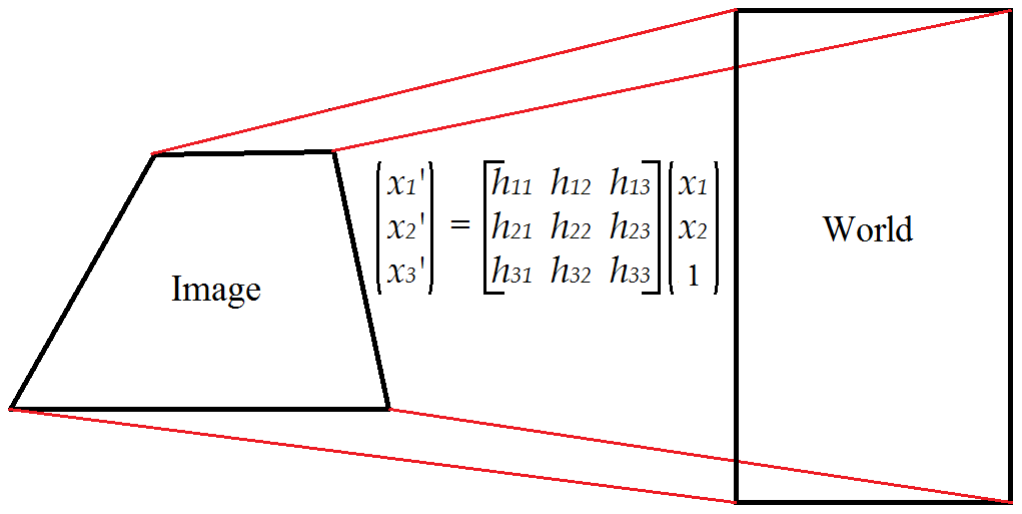
Plane to plane mapping which transforms points in one plane to points in the other plane using the homography matrix.

In order to estimate the position of the players on the field, the first step is to map the pixel positions of a user selected calibration image into real world coordinates. This is done by calibrating the field by selecting the four corners of the field in the image, and assigning each corner its corresponding coordinates. The coordinate system's center is at the bottom left corner of the field (0;0). The remaining three corners at (0;8), (8;16) and (0;16) get assigned their corresponding image coordinates in pixels. The mapping of the image plane into the real world ground plane is done through a linear projective transformation (homography), as seen in Figure 1. For details, please refer to [15].

All the calculations and position estimations are, if not otherwise noted, done in real world coordinates in metric units. Given the camera perspective, this means a higher resolution in the front of the field than in the back, where the objects become smaller.

#### b) Background subtraction

One of the most important aspects of the used tracking method is the background subtraction. The idea behind it is that we want to recognize foreground objects, likely to be the players, which constitute the pixels differing from the static background image. Based on the Gaussian Mixture Model explained in [16] we first calculate a background using 16 Gaussian modes, taking every *N-th* frame to feed the model. Since this model is recursive and the resulting background image is based on a given number of input frames, we found *N = 10* to be a good number, allowing the movement of the players between consecutive input frames to the model to average out. The resulting background image is further recursively fed back into the model as every 3^rd^ input, thus giving the models' memory a cleaner image allowing for more stable backgrounds and preventing the players becoming part of the background. By adaptively changing the models' learning rate when considerable changes in the scene occur, such as involuntary camera motion or sudden illumination changes, we make new data for the background model more influential to quickly adapt to these changes. Thus, we constantly monitor the number of foreground pixels in the image (as explained in the following section) and when the count exceeds normal values, the learning rate is set higher. When the count reaches normal values again, the learning rate is lowered again for a more stable background calculation.

#### c) Foreground mask

**Figure 2:**
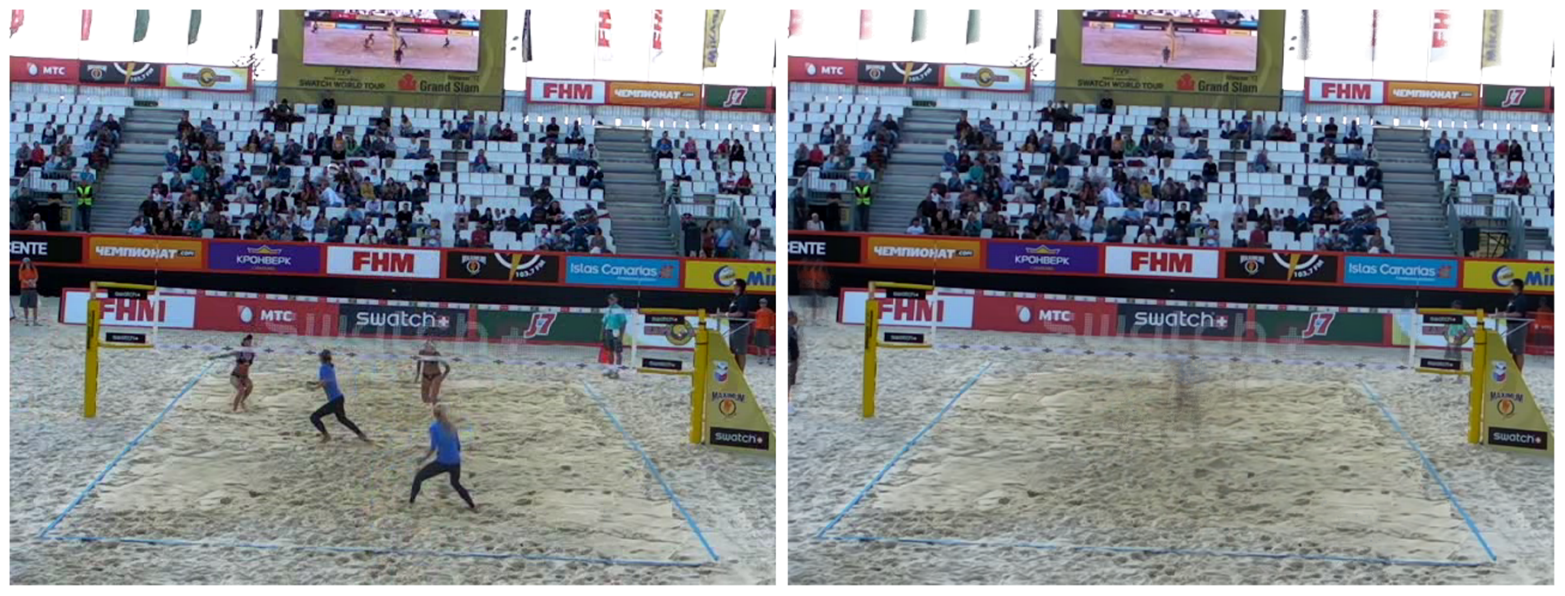
Exemplary frame from a video (left) and the background calculated for this scene (right).

**Figure 3:**
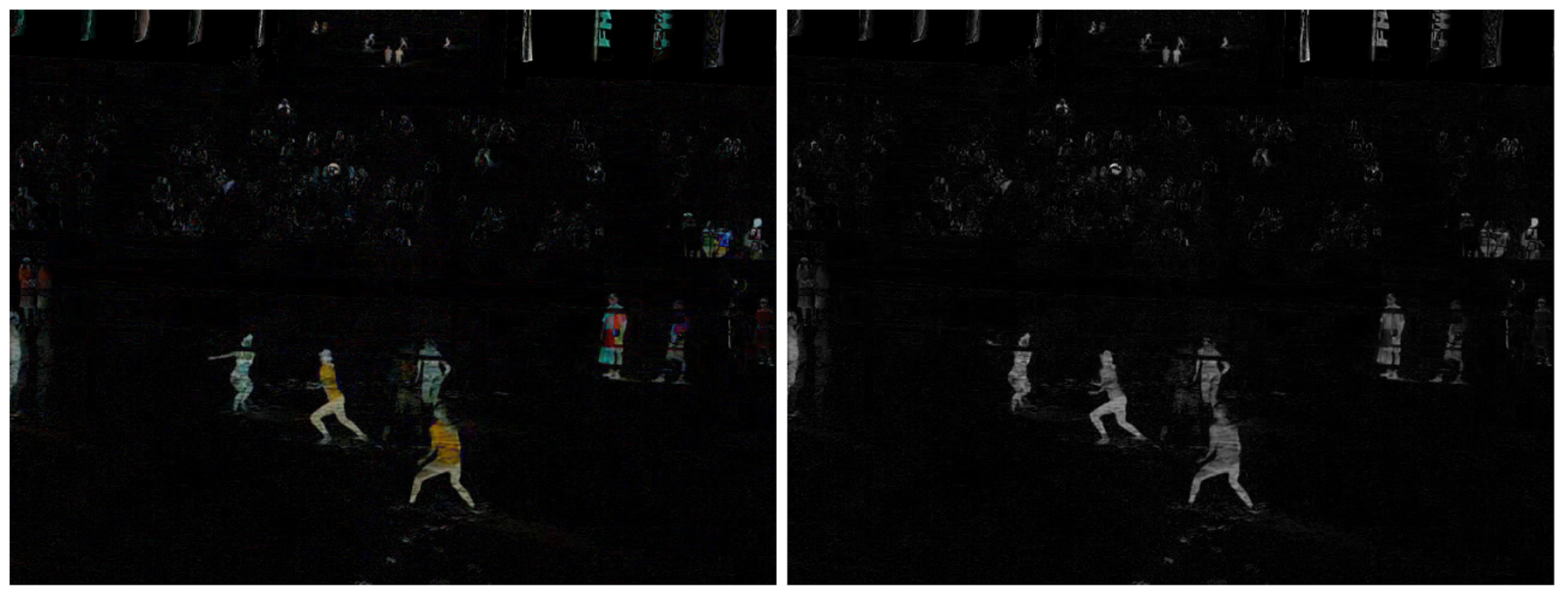
Difference image obtained from subtraction of the background image from the current frame (left), and then single channel grayscale converted (right).

**Figure 4:**
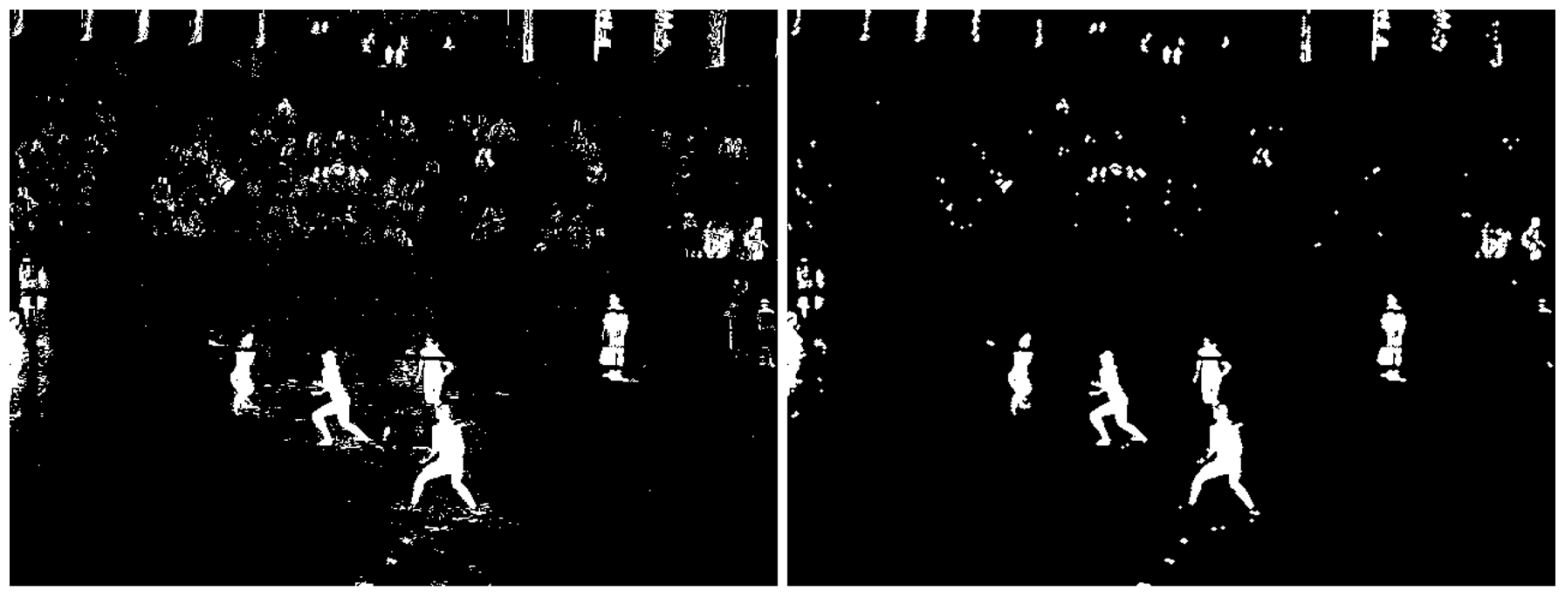
Binary image after thresholding (left) and result after applying the opening filter (right).

**Figure 5:**
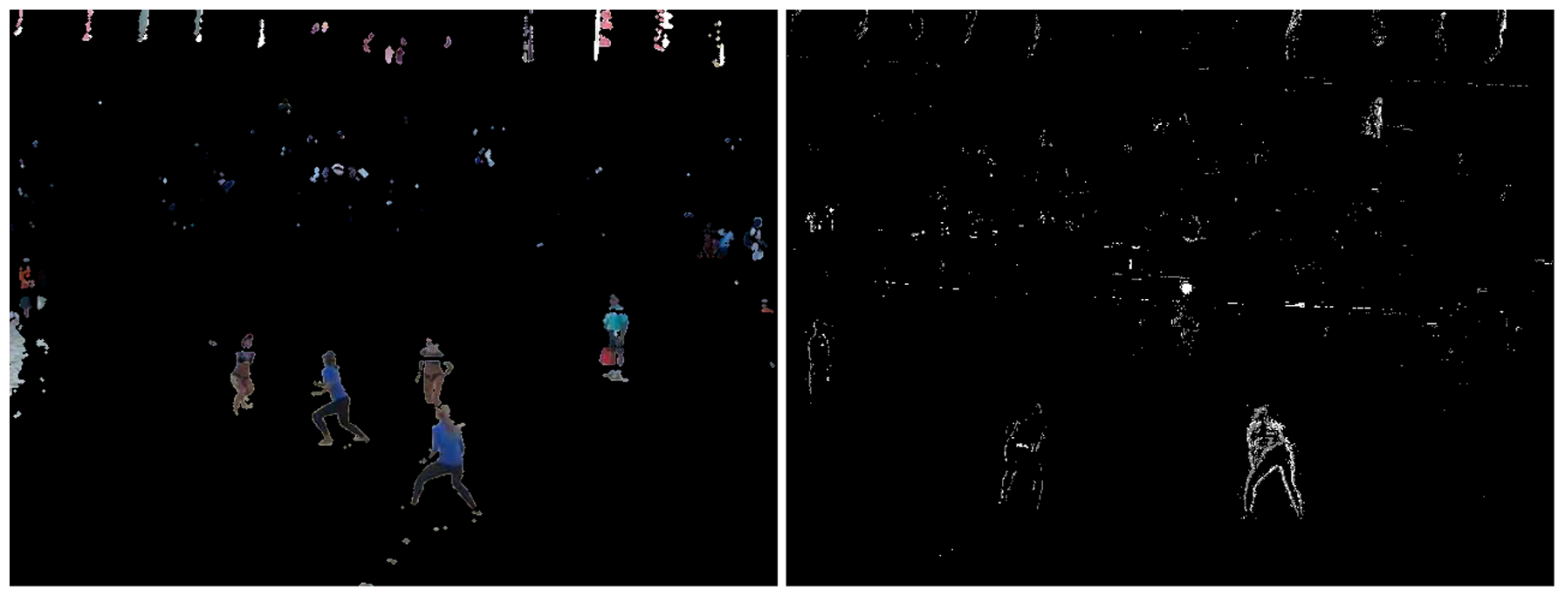
Final result after applying the foreground mask to the current frame of the video (left) and binary image showing pixels differing from the previous frame and used for calculating the movement weight for each particle (right).

A second processing step in the model is the foreground mask extraction using the background subtraction described in the previous section. The purpose of the foreground mask is the elimination of unwanted background color information in the tracking. As the texture of the field changes constantly due to wind or footsteps of the players in the sand, its color information changes as well. Also, objects with similar colors as the players' clothing, hair or skin can be left out of the tracking as to not confuse the particle filters. As shown in Figure 2, first the calculated background image is subtracted from the current frame. The obtained difference image is then converted into a 1 channel grayscale image (Figure 3), which is further thresholded to obtain a binary black and white image of the scene. A simple opening morphological filter is then applied to reduce noise, mainly from the spectators and the sand (Figure 4). Finally this resulting mask is applied to the original current frame, leaving only the foreground objects in color, while the background becomes zero valued (Figure 5 left). On these preprocessed images containing only foreground information we apply our two tracking methods: The classical particle method and the integral histogram method. Both methods use the weighting algorithms for the particles' weights as explained in sections 3.1d–f.

#### d) Mask weighting

**Figure 6:**
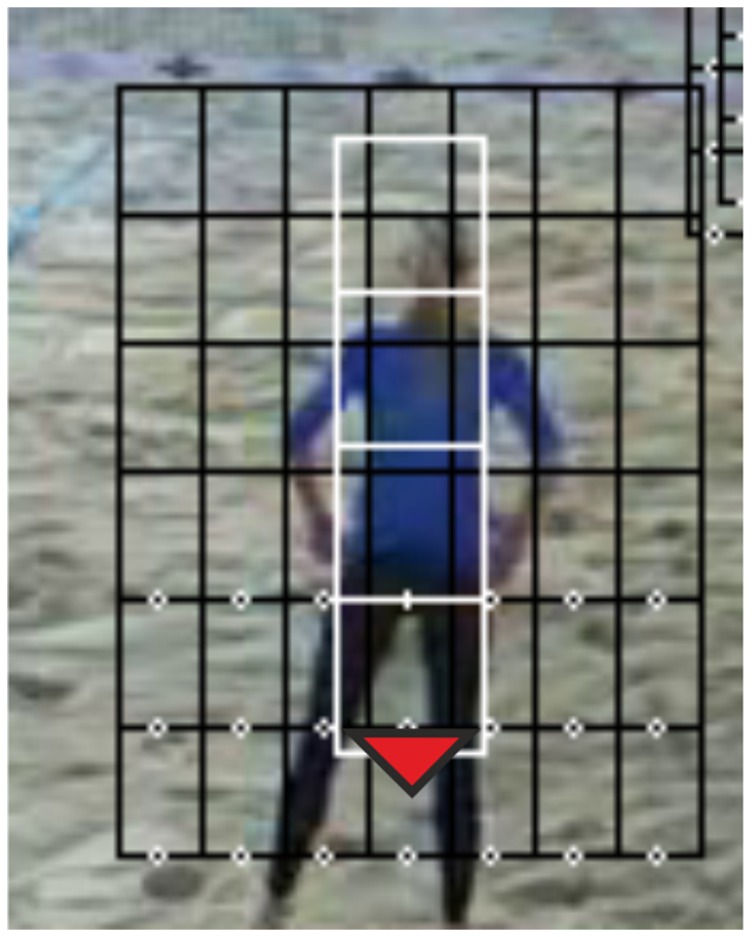
Screenshot of the tracking of one of the players by means of the integral histogram approach. The grid is shown on the original frame for illustration purposes, while the tracking is actually done on the foreground mask filtered frame to suppress the background from the calculations.

For the prediction of a player's position on the field one powerful cue is the presence of foreground objects, since only foreground objects can possibly be the players. The foreground mask as described above (shown in Figure 4 on the right side) gives good information on whether a pixel in the image corresponds to a player or not. As described in [14], for each player a particle set of 20–50 particles is defined and a squared bounding box which can be subdivided into subwindows assigned to each particle (e.g. see Figure 6, where the red triangle represents the mean particle in a set and the 4 white squares represent this mean particle's subwindows). For each particle the number of non-zero pixels of the foreground mask is counted in each subwindow of the bounding box and normalized to its width and to the maximum count for all particles in that frame. This then gives a normalized weight to each particle, which is related to the probability of the particle state being the position of a player. Thus, when a particle is close to the real player's position, the non-zero count will be high and the corresponding weight high as well, while the weight of a particle positioned somewhere else in the field (probably on the black parts of the foreground image) will have a much lower count and therefore a low mask weight.

#### e) Movement weighting

The second cue for the tracking of the players is the movement between consecutive frames, given that the camera is held static. While some structures or referees may contribute to the foreground mask weighting, it's usually only the players who move on or around the field. Therefore, similarly to the foreground mask weight, a new weight is defined that only takes into account the change of pixels between consecutive frames, related to players movement. For this, a binary image showing only the pixels that differ from the previous frame is created using the background subtractor introduced in section 3.2 and in the same way as explained for the mask weight (section 3.1.d), the non-zero pixels are counted for each subwindow of a particle, assigning a movement weight to each. An exemplary binary image generated from movement is shown in Figure 5 (right), where the ball is clearly visible as a bright point in the middle of the image, as well as the two receiving players in the front. All objects that are static will not appear in this binary image. This is a good thing for the particle weighting, since there is a larger possibility of a particle set to lose a player if the player moves fast around the field. Any kind of movement will usually alter the color information (e.g. if the player moves from shady to sunny regions). Thus, this movement weight will increase the weight of a particle that has inside its subwindows many non-zero pixels due to movement, and therefore help to fix the tracker to the tracked object or player, especially when the whole object is moving.

#### f) Color weighting

The color weighting is the most important of the three weights, since it is the only cue that allows a differentiation between individual players. First, for the user selected calibration image a foreground mask image such as shown in Figure 5 (left) is created. As the user enters the position of the players in the calibration image (by selecting a frame with the players in an upright position and clicking between their feet), a particle is created for each of the four positions and the color histogram calculated for each subwindow of the particle's bounding box. Similar to the method described in [17] we took the HSV color space and created a one dimensional histogram containing **N_H_^
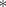
^N_S_** bins and **N_V_** bins appended. This histogram gives a unique “fingerprint” for each subwindow, and is taken as reference histogram for later comparisons in the frame to frame calculations. Contrary to calculating color histograms on the original frames, using the foreground mask directly reduces greatly the possible particle states, thus giving a more accurate predicted players state and avoiding confusions with similarly colored structures of the background. Also, as proposed by [17] and implemented in [13], [14], using the subwindows to include a spatial dependence between them helps to place the particles' positions between the feet of the players, as originally entered by the user in the calibration image. We couple the upper subwindows tightly by multiplying the individual subwindow-color-weights linearly, but leaving the lowest subwindow loosely coupled by just adding its color-weight to the total color-weight, since it usually only contains the lower legs. The lower extremities of the players experience most of the movement with the legs often wide spread, in which case the lowest subwindow may not include the legs' information at all. The histograms for each particle in every frame are correlated with the reference histograms extracted from the players in the beginning by means of the Bhattacharyya distance:
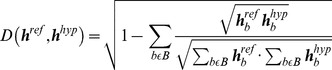
(1.1)Where ***B*** denotes the number of histogram bins (**N_H_^
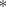
^N_S_+N_V_**), and ***h_b_*** represents the value of the ***b-th*** bin of the reference or hypothesized histogram. The smaller this distance, the more likely it is that the respective particle's state corresponds to the player's position [14]. Depending on the number of bins, changes in illumination or color will influence the color weighting more or less. When choosing a greater bin number for the histogram, small changes of the H, S or V value may lead to the pixel being counted to a different bin (increasing the Bhattacharyya distance), while choosing smaller bin values leaves more space to changes in the image and reducing computational cost, but making the differentiation between players of the same team less accurate. We found a good compromise to be **N_H_ = 10**, **N_S_ = 10** and **N_V_ = 10**, resulting in a 1-D histogram of 110 bins (see the results section for a comparison of different bin sizes on the tracking results).

#### 1.1 Particle filter method

Two different tracking algorithms were implemented in this work, both including the weighting methods described above. The classical particle filter algorithm assigns one particle set to each of the four players. If there is information available from a pre-structuring step, such as the side of the field where the teams are located or the serving team, the particles of each set will be randomly positioned in a selected area of the field which is likely to contain one of the team's players. Elsewise, we search the entire field for players. We use the particle filter as described in [17] and modified to our weighting system. For the resampling of the particles at every new frame we use the roulette-wheel resampling algorithm described by [18], choosing a random Gaussian noising with a standard deviation of 0.2 meters. An additional modification is that the particles with a weight in the upper 0.1% of the maximum weight of a set will be resampled with only 0.04 meters standard deviation. Using the roulette-wheel resampling, particles are resampled to higher weighted particles' positions of the previous frame with more probability, and by adding random noise in the resampling process an area around the most probable states is covered. The particle positions are always calculated in world coordinates as given by the homography transform described in section 3.1.a. For every particle in each set the mask, motion and color weights are calculated and summed as given in (2–5).
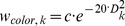
(1.2)

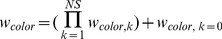
(1.3)

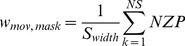
(1.4)

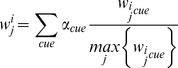
(1.5)With ***c*** being a scaling constant to avoid too small weights (we used a value of ***c = 100***), ***D*** the Bhattacharyya distance defined in equation 1, ***NS*** the number of subwindows of the rectangular area corresponding to one particle, ***S_width_*** the width of the subwindows for normalization purposes, ***NZP*** the number of non-zero pixels in the movement or foreground mask binary image counted in the ***k-th*** subwindow. The total weight of the ***j-th*** particle corresponding to the ***i-th*** player is the sum of the individual cues: color, movement and foreground mask. Each cue weight is normalized to the maximum cue weight of the ***i-th*** set for the current frame. The weighting constant **α** is cue dependent and gives best results for **α** ***_color_ = 1***, **α** ***_mask_ = 0.4*** and **α** ***_mov_ = 0.1***. The estimated position of each player is the weighted average of the states of the individual particles' of a set, giving higher weighted particles more importance for the estimate. To ensure that a particle set does not get confused and sticks to another player, we implemented a safeguard such that if two clouds track the same player for more than ***10*** frames, the cloud with the smaller average state color weight will be resampled uniformly in the field to find the lost player again.

#### 1.2 Integral histogram method

As used by [13], we also implemented an integral histogram approach for comparison with the classical particle filter described in the previous section. Proposed by [19], the computation of histograms using the integral histogram approach can be done in a faster way than calculating a great number of individual histograms. Since the areas covered by a sets' particles often overlap, the integral histogram calculation may be faster if the number of particles in a set is greater than the number of individual histograms of the grid in the integral histogram approach. The two approaches differ greatly in their distribution of particles, as in the classical particle filter the particles are resampled to a random cloud around the most probable states, while in our integral histogram approach we keep a rigid distribution of the particles in the grid to speed up the calculations. As can be seen in Figure 6, the grid (in black) is a defined area around a player that is partitioned into e.g. 6 rows and 7 columns with the particles distributed at the lower three rows. The red dot represents the predicted state taken as the weighted average of the individual particles' states of the set. The new position of the grid in the next frame will be adjusted to this prediction.

As in the particle filter in the previous section, the area of the grid is adjusted using the homographic transform, adapting to the perspective size change of the players with respect to the distance from the static camera. The individual particles in the grid are weighted as in the particle filter method with the color, movement and foreground mask cues. Additionally, the particles are weighted by their position in the grid, such that the particles in the center columns are higher weighted than the ones in the outer columns by a quadratic factor. This is done to avoid confusion with another player with similar colors when he or she comes near the tracked player. As in the particle filter method, two sets tracking the same player for more than ***10*** frames will lead to a resampling of the weaker set.

### 2. Ball tracking

**Figure 7:**
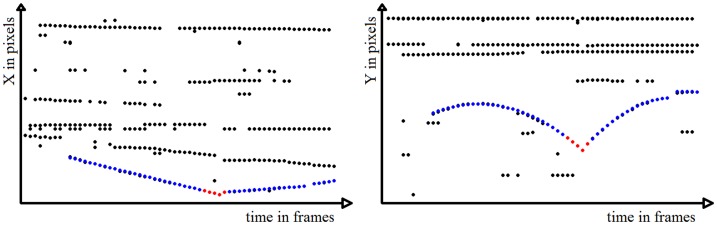
Horizontal candidates positions plotted over time (left) and vertical candidates positions plotted over time (right). Matching candidates on both relationships are shown in blue, interpolated candidates in red.

One of the most important aspects for statistical video analysis in beach volleyball and many other sports is the position of the players at the ball contact time points. Tracking of the ball is a challenging task as color and shape cues are seldom good, since the colors of the ball get blurred due to its spin and velocity, and the roundness of the ball is hardly distinguishable when in motion at a standard 25 frames/second video. We developed a separate tracking system for the ball in order to automatically detect these time points from the ball trajectories. In [20], a ball tracking algorithm was proposed that promises a ball tracking rate of around 90%. We adapted this algorithm to our framework and further developed an alternative ball tracking algorithm based on the Hough line detection for comparison. Both algorithms use the binary movement image described in the movement weighting section (3.1 e). We search for blobs in the frame having an area in a range defined by the actual ball size (21 cm diameter) and taking into account the homographic transformation of the field to adapt to different scene scalings. From these blobs we look for ball candidates by defining two concentric squares of sizes *40×40* and *60×60* pixels for the inner and outer square respectively. Our restriction for the ball candidates is for the inner square to hold more than Q non-zero pixels, while the outer square may only hold Q+R non-zero pixels. By keeping R low (e.g. 5 pixels), we ensure that the candidate is an isolated blob. Blobs that match both criteria are saved into memory and the process is repeated for subsequent frames. From the physical ball trajectory motion we know that the ball motion in the horizontal direction follows straight paths, while the vertical direction follows parabolic paths. The next step is the generation of trajectories. The adapted algorithm from [20] initializes a trajectory when at least three candidates with a distance between them of less than 20 pixels form a straight line in the X-distribution image. The new trajectory is generated by equations 6 and 7.
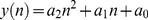
(1.6)

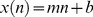
(1.7)Where ***a_2_, a_1_, a_0_, m, b*** are real valued constants and ***a_2_*** *<0*. Using these equations the next ball position is estimated and compared to the next ball candidates. If one of the candidates is close to the estimated position it is added to the trajectory and the equations updated. If no candidate is near the estimated position the frame is denoted as missing frame. A trajectory is terminated if there are more than three missing frames. Of the trajectories that occur parallel in time we select the ones with the lowest deviation from the equations and with the higher number of candidates, ensuring that only one trajectory be present at a given time. Finally, as our main interest is in finding the intersection of the trajectories giving the contact time points, we integrate the trajectories by extending the individual ones based on their equations, as can be seen in Figure 7 in red.

**Figure 8:**
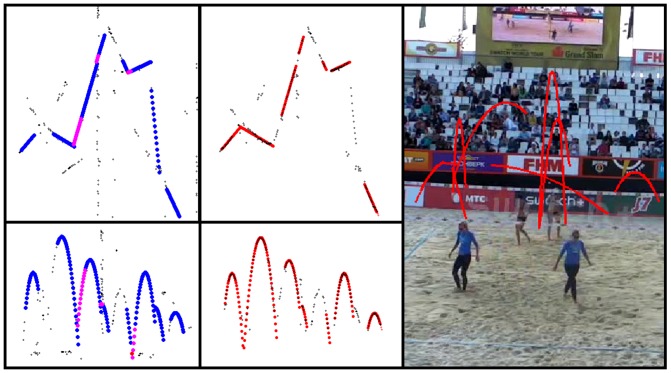
Candidate trajectories found by the algorithm as proposed in [20] (left) are shown in blue, with interpolated values continuing a found trajectory shown in magenta. The Hough based algorithm results are shown in the middle and right for an exemplary video sequence with 8 ball contacts.

The second algorithm based on the Hough line detection takes the ball candidate image (Figure 7: the black points are the ball candidates) and finds lines in the x-distribution image and y-distribution image that are horizontal. Points lying close to these lines are disregarded. For non-horizontal lines found in the image of the remaining candidates with a minimum length of 20 pixels and a maximum gap between points of 10 pixels, we assign those candidates that lie close to a line to its corresponding line. For the candidates corresponding to each line we determine the slope ***m*** and zero crossing ***b*** that fits these candidates best, and do the same for the parabolic parameters ***a_2_***, ***a_1_***, and ***a_0_***. We then create new trajectories for each set of parameters in the range of the smallest and largest frame of the line candidate set. Neighboring line sets are joined by interpolation if their line intersection lies between the maximum frame of the previous line set and the minimum frame of the current line set, and if the Euclidean distance between the y values of both sets at the intersection frame is smaller than 15 pixels. These restrictions are necessary since in some cases the parameter set is erroneous and we only want to interpolate if the trajectory candidates intersect where supposed to. Figure 8 shows the trajectories found by the algorithm proposed in [20] (left) and by the Hough based algorithm (middle) for an exemplary video sequence. On the right the final trajectories found by the Hough based algorithm are depicted in red on the final video frame. Results of the algorithm proposed in [20] can be seen in the exemplary Video S1.

In order to determine the ball contact frames, we save the frame numbers of the intersections of the y-distribution trajectories into a file for later comparison with reference contact frames extracted manually. We choose the y intersections of the parabolic trajectories since it gives more accurate intersection frames than the x intersections of the lines. The first ball contact corresponding to the serving cannot be determined by trajectory intersection. Thus we take the initial frame of the trajectory as the estimated 1^st^ ball contact frame.

## Evaluation and Results

### 1. Tracking of the players

We evaluated our tracking algorithms for the different tracking methods separately. For the tracking of the players different videos of female and male players were used and the results of our estimated positions compared to manually annotated positions. Our restriction for a correct tracking was defined such that the estimated position differed from the reference position by less than 0.5 meters or less than 20 pixels, the latter playing a greater role for the players in the back of the field due to the perspective view. We used 12 pre-structured videos of an average length of 259 frames holding information on the beginning and ending frame of a point. A new calibration was used for each new game, but the same calibration was kept for different game points of the same game, therefore keeping the same reference histograms for the players. Our algorithm automatically searched for the players on the field at each new game point and a new comparison was started when all four players were correctly tracked for at least two consecutive frames. Then, we compared the tracking results with the reference positions at every ***3^rd^*** frame until the ending frame of a game point. We compared different parameter settings, varying on the one hand the number of particles in a set with values of ***30*** and ***50*** particles for the particle filter, and ***15*** and ***33*** for the integral histogram method. Additionally we varied the number of histogram bins ***N = N_H_ = N_S_ = N_V_*** to the values ***5***, ***10*** and ***15*** to analyze the effect of the number of bins on the tracking. These different bin numbers N give 1-D histograms of size **B =** ***30***, **B =** ***110*** and **B =** ***240*** respectively as explained in section 3.1.f with **B = N_H_^
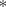
^N_S_+N_V_**. Since usually players in the same team have very similar colors, we allowed switching of the particle sets between players of the same team to allow better comparison of the results with lower bin sizes, where more confusion between players can happen due to the coarser bin quantization. The tracking results for the particle filter method are shown in Table 1.

**Table 1:** Results for the tracking of the players – Particle filter method.

	P:30, N:5	P:50, N:5	P:30, N:10	P:50, N10	P:30, N:15	P:50, N:15	Average
P. Front 1	79,0	74,9	73,3	61,0	79,9	66,5	72,4
P. Front 2	79,0	88,2	66,7	76,1	78,1	72,3	76,7
P. Back 1	59,7	54,9	63,6	56,6	56,5	56,6	58,0
P. Back 2	61,7	50,6	65,3	63,4	62,9	54,3	59,7
P. Front Av.	79,0	81,5	70,0	68,6	79,0	69,4	74,6
P. Back Av.	60,7	52,8	64,4	60,0	59,7	55,4	58,8

Average results for the particle filter method for particle set sizes of P = 30 and P = 50 particles and bin numbers N = 5, N = 10 and N = 15. A total of 12 videos of average length of 259 frames were evaluated at every 3rd frame.

For the integral histogram method we used 15 and 33 particles, which were arranged in 3 rows (as shown in Figure 6) resulting in grids of 5 and 11 columns respectively. The difference between both grids is solely the distance between the particles in the horizontal dimension, but keeping the total area of the grids the same. The average results for this method are shown in Table 2.

**Table 2:** Results for the tracking of the players – Integral histogram method.

	P:15, N:5	P:33, N:5	P:15, N:10	P:33, N:10	P:15, N:15	P:33, N:15	Average
P. Front 1	79,6	77,4	84,1	87,1	85,0	78,8	82,0
P. Front 2	88,2	85,9	86,1	79,1	86,4	73,9	83,3
P. Back 1	50,8	44,4	53,4	45,6	57,2	47,4	49,8
P. Back 2	73,5	72,4	74,9	77,3	73,9	67,7	73,3
P. Front Av.	83,9	81,7	85,1	83,1	85,7	76,3	82,6
P. Back Av.	62,2	58,4	64,2	61,4	65,5	57,6	61,6

Average results for the integral histogram method for particle set sizes of P = 15 and P = 33 particles and bin numbers N = 5, N = 10 and N = 15. A total of 12 videos of average length of 259 frames were evaluated at every 3^rd^ frame.

### 2. Tracking of the ball

For the ball tracking algorithms we evaluated both mens and womens volleyball matches at different illuminations and perspectives. We used 7 different videos with more than 4 ball contacts evaluating 535 total frames in average taken at every 3^rd^ frame from the videos. Reference ball positions were manually annotated. The tracked ball was marked as correctly tracked if the Euclidean distance between the reference point and the tracked position was less than 20 pixels. The results are summarized in Table 3.

**Table 3:** Results for the tracking of the ball.

	Total frames evaluated	Frames tracked	Correctly tracked frames	Correctly tracked frames in %
Chen [20]	553	337	300	54,2
Hough lines	517	268	218	42,1
Ball contacts	190	107	73 intersections+20 first frames	48,9

Results for the tracking of the ball using the algorithm proposed in [20] and the algorithm based on the Hough line detection. The last row shows the result of the estimated ball contact points.

### 3. Contact time point estimation

For the determination of the ball contact frames we evaluated 31 videos with a total of 190 ball contacts. We used the y intersection frames of the algorithm proposed in [20] and the initial frames of the 1^st^ trajectories as ball contact estimates. Reference ball contact frames were manually annotated. A ball contact frame was marked as correct if the frame number differed in less than 10 frames from the reference ball contact frame. The results are summarized in Table 3.

### 4. Time efficiency of the tracking methods

In this work, we used a laptop with an Intel Core i7-2820QM CPU at 2.30 GHz and 8 GB of RAM space from the year 2011. Our code was not specifically optimized for fastest performance but rather for good integration into the tracking framework BeachTracker and easiness to develop and test our algorithms. The code was written in C++ and run using a GUI written in Qt. The average time needed for the tracking of the players normalized to 1 second of video material is given in Table 4. For the tracking of the ball, the time needed for either of the two methods was less than the time used for playing the video, thus it can be considered real time.

**Table 4:** Results for the time efficiency of the algorithms.

Particle num. P, Bin num. N	Time in sec for Particle Filter	Particle num. P, Bin num. N	Time in sec for Integral histogram
P: 30, N: 5	5,67	P: 15, N: 5	2,41
P: 30, N: 10	5,83	P: 15, N: 10	2,63
P: 30, N: 15	6,00	P: 15, N: 15	2,50
P: 50, N: 5	8,67	P: 33, N: 5	4,13
P: 50, N: 10	8,75	P: 33, N: 10	4,17
P: 50, N: 15	9,17	P: 33, N: 15	4,42

Results for the average time needed for tracking of the players normalized to one second of video material at 30 frames/second and a resolution of 640×480 pixels. Results are given for varying particle numbers P and number of bins N.

## Discussion

The results presented in the previous section will be discussed in this section in detail. Starting with the classical particle filter it is visible from Table 1 that a greater number of particles does not increase the tracking accuracy as might be expected, but on the contrary decreases the tracking accuracy. This may be due to an increase in confusions with other players, since with greater number of particles also the probability of a split of the cloud increases. As the predicted state is the average of the individual particle states of a set, when a split occurs the predicted state will lie in between the splits. As we allowed swapping of the players of the same team in the evaluation it is not possible to discuss the influence of the bin sizes on the tracking of individual players. Yet for this setting it seems favorable to use a small number of bins since the results are similar to those with higher bin sizes with the advantage of less computation time. Table 2 shows the results for the integral histogram tracking method. Here we can also see a better tracking accuracy for smaller particle numbers. The difference between smaller and higher particle numbers is only the horizontal density. Since the players are mostly bent, jumping or running while a point is being played it might be that increasing the horizontal density biases the predicted state to a different equilibrium position than between a player's legs which was the reference player's position. No definite conclusions can be made on the influence of different bin sizes on the tracking accuracy, although a tendency can be seen in the results where a bin number of 10 is on average better than 5 or 15. This is especially evident for the players in the back for both the classical particle filter method and the integral histogram method. For the tracking of the players we can observe a clear superiority of the integral histogram method over the classical particle filter. This is very good since not only are the tracking results better by 6% for the players in the front and 3% for the players in the back, but it also uses less resources and is therefore computationally much faster as can be seen in Table 4. The discrepancy between the average tracking results of the players in the back of 23,5% for the integral histogram method versus 1,7% for the classical particle filter using the same calibrations for both methods yet different calibrations for different videos can only be explained by the difference in area covered by both approaches. For the particle filter we only have a cloud spread of a couple of pixels in the back while the integral histogram method covers many times that number. This result suggests a better discrimination of individual players by the latter method, meaning less confusion between players of the same team, regardless of the histogram bin count. Comparison of the tracking results of this work with the previous work in [14] apparently does not show any improvement. Yet in [14] we had much longer tracking situations of static scenes. In this work however we used pre-structured videos that only showed the time from the serving to the end of the point, meaning much more percental movement in the scene than in continuous videos. The great importance of using the foreground mask to get rid of the background in the calibration image, and thus in the reference histogram, is evident when considering the spatial dependency of the subwindows of a particle. If the background were not removed, the net would be included in the back player's reference histogram as false color information, and also in the spatial relationship of the subwindows, thus leading the algorithm to expect the net always at the same height (or in the same subwindow as in the reference histogram) of a back player. This would be erroneous and should be considered also for other tracking applications with spatial relationships when occlusions by static objects are present in the calibration. The advantage of using a foreground image for the reference histograms is evident if any kind of occlusion of a tracked object is present during calibration. For the ball tracking we obtained very different results than the ones presented in [20], where our results strongly showed that the tracking accuracy claimed by the authors was not achievable under realistic conditions. Although we optimized the algorithm to our framework we could only track little more than half the balls in the game, compared to over 90% as presented in their work. Although we could track over 80% of the balls in certain favorable video scenes (as can be seen in Figure 8) this algorithm does not deliver the stated accuracy when used in real situations. Yet it performed better than our simpler algorithm based on the Hough line detection. We believe that the filtering of noise from the ball candidates' image by removing all ball candidates close to horizontal lines also removes several ball candidates that belong to a trajectory. While the noise removal results in very clear images with almost only candidates belonging to a trajectory visible, it also removes crucial information needed for detecting trajectories. The results of the ball contact frames are similar to the ball trajectory tracking accuracy. When the ball tracking performs well the number of correct ball contact points is also high as would be expected since a higher number of detected trajectories leads to a higher number of trajectory intersections. We think that by placing the camera at the side of the field rather than at the back of the field could drastically increase the tracking results of both the players and the ball since there would be much less mutual occlusions by the players and the net, and a wider area covered by the camera perspective. The algorithm code used here is given in Code S1.

## Conclusions

In this work we developed methods for the tracking of the players and the ball in beach volleyball videos. For the players' tracking we used three different cues, namely foreground mask, movement and color cues. For the ball tracking we only used motion information between consecutive frames based on a background subtraction algorithm. We compared two different tracking methods for the players' tracking and two different methods for the ball tracking. The integral histogram method clearly outperformed the classical particle filter method and further performed best with a smaller number of particles. The ball tracking method from [20] performed better than the Hough line detection algorithm, but with only 54% tracking accuracy by far did not reach the promised tracking results of over 90%. Higher tracking rates could possibly be achieved by improving the video capture (e.g. angle, camera position, higher resolution) and by combining this method with other successful tracking methods, e.g. as proposed in [10]. Improvements for future work include the usage of higher definition videos that allow a better player discrimination, the placement of the camera at the sides of the field and better algorithms to prevent player confusion. Also, a confidence measure of the tracking certainty could be delivered by the algorithm automatically marking those frames where manual correction is necessary. One possible confidence measure could be the distance of a predicted state from the other three states, such that if the distance of one tracked player is far apart from the other players' positions, a higher confidence measure would be calculated since there would be less confusion probability. As a second confidence measure, the cloud spread of a particle set could be taken which would be smaller if the predicted state was very probable since the majority of particles would be centered at the correct tracked object. Also, this framework could be adapted without much effort for different sport applications such as other ball sports and even for video surveillance purposes. The proposed tracking algorithms of this work could be tested in diverse fields and compared to existing tracking methods as proposed in [21]. In addition, the challenging tracking scenario of beach volleyball could be included in the benchmark framework of [21] to be able to test the performance of different trackers in tracking fast moving players and the ball.

## Supporting Information

Code S1 **Algorithm code.** The algorithm code used in this work for tracking the ball and the players.
(ZIP)

Video S1 **Ball tracking video example.** An exemplary video of the ball tracking algorithm from [20].
(AVI)
